# Supplementary material for: The Autocatalytic Chemical Reaction of a Soluble Biopolymer Derived from Municipal Biowaste
Source: Molecules. 2024 Jan 18;29(2):485. doi: 10.3390/molecules29020485 (PMC10818964; doi:10.3390/molecules29020485)
Supplement: Supplementary file 1 [file molecules-29-00485-s001.zip › molecules-2786408-supplementary.pdf]

Table S1. Experimental conditions and products for the oxidation[a,b] of SBP in water solution at 33 g/L concentration, irradiated and not irradiated with simulated solar light, in the absence and presence of added hydrogen peroxide at 2-3 H_2O_2/C mole ratio, with and without pH control[c,d]. Data for pristine SBP at day 0 and recovered soluble and insoluble products at day 14[e]: weight (WY, w/w %), and C (CMY) and N (NMY) molar (mol/mol%) yields relative to pristine SBP[f], and organic C and N w/w % content[g] in recovered materials.

Treatment[b]		pH[c]	KOH[d]	H^+/C,[d]	Product[e]	Yields (Y)[f]			Content %[g]		C/N w/w
			grams	eq/mol		WY	CMY	NMY	N	C	
Nr.	type										
0	None	10			SBP				4.14	35.83	8.7
1	D0	9			soluble	73.9	79.3	80	4.50	38.69	8.6
					insoluble	3.8	2.9	3.3	3.58	28.8	7.9
					total	77.7	82.2	83.3			
2	D2	5.3			soluble	71.2	58.2	111	6.47	29.41	4.5
					insoluble	7.2	4.4	3.1	1.81	21.9	12.1
3	D3	4.9			soluble	54.8	36.8	95	7.17	23.84	3.4
					insoluble	12.4	7.8	8.4	2.80	22.96	8.2
4	L0	9			soluble	85.2	91.8	91	4.44	38.77	8.8
					insoluble	3.4	2.9	3.4	4.10	30.34	7.4
5	L2	4.9			soluble	65.4	53.9	109	6.91	29.68	4.3
					insoluble	13.8	8.9	8.4	2.51	23.3	9.3
6	L3	5.1			soluble	51.4	35.1	96	7.70	24.47	3.2
					insoluble	15.4	9.4	6.9	1.86	22.1	11.9
7	D0	10	2	0.24	soluble	88.5	100	88	2.93	27.89	10.2
					insoluble	4.3	3.8	3.6	2.50	19.20	9.1
8	D2	10	2.8	0.33	soluble	97.4	94.1	77	2.10	19.09	10.6
					insoluble	4.4	3.9	1.7	1.06	19.72	19.3
9	D3	10	3.3	0.40	soluble	97.7	90.1	70	1.77	16.71	11.2
					insoluble	4.8	4.7	4.1	2.10	20.65	10.0
10	L0	10	2	0.24	soluble	95.0	100	92	2.87	27.08	10.1
					insoluble	5.3	4.3	3.7	2.07	16.44	10.1
11	L2	10	2.9	0.35	soluble	95.9	84.7	67	1.82	17.06	11.0
					insoluble	4.9	4.4	3.2	1.72	19.76	11.9
12	L3	10	3.3	0.40	soluble	107	98.4	74	1.74	16.92	11.5
					insoluble	5.2	4.8	3.5	1.71	19.21	11.6

[a]See section 4 for details. [b]Nr (treatment number), D (dark, no irradiation); L (simulated solar light irradiation); Di and Li, i = 0, 2-3 H_2O_2/C mole ratio. [c]Final pH after 14 days treatment; starting pH was 10 at day 0 for all treatments. [d]Added KOH (g) to keep pH 10 constant and corresponding acid equivalents produced per pristine organic C mole (H^+/C eq/mol). [e]Pristine SBP at day 0, and recovered soluble and insoluble product at day 14). [f]WY, CMY and NMY (% weight, molar C and N yields, respectively, relative to pristine SBP). [g]Weight % content of C and N in product. Data for treatment 0-none are for the pristine SBP solution at day 0

Table S2. C mole % recoveries[a] accounted for by molecular weight fractions (MWF)[b] isolated by sequential membrane ultrafiltration in the SBP treatments[c] listed in Table S1.

MWF[b]	1-D0	2-D2	3-D3	4-L0	5-L2	6-L3	7-D0	8-D2	9-D3	10-L0	11-L2	12-L3
R750	4.2	13.5		4.0			30.0	7.9	12.6	0.5	4.3	10.0
R150	46.0	20.1	1.0	1.9	5.0	0.7	6.5	8.6		0.5		
R50			0.6				1.3	0.5		14.2	5.7	
R20			0.8	68.7		0.9	11.6	6.6	3.0	33.5		12.1
R5				0.1		2.0						2.7
R0.2			7.2	0.1	41.3		11.7	16.0	16.0	17.8	1.2	19
P0.2	28.3	28.6	26.9	10.4	3.4	26.0	14.8	24.7	30.1	11.4	34.7	22.2
P0.2+R0.2[d]	36	46	93	12.3	89.9	87.8	34.9	63.2	74.7	37.4	78.2	62.4

[a]Data are referred to the carbon in the pristine SBP yielding the products in the different treatments in Table S1. [b]Ri (i=750-0.2) retentates and P0.2 permeate fractions containing molecules in the following molecular weight ranges: over 750 kDa for R750, from 750 to 150 kDa for R150, from 150 to 100 kDa for R100, from 100 to 50 kDa for R50, from 50 to 30 kDa for R30, from 30 to 20 kDa for R20, from 20 to 5 kDa for R5, from 5 to 0.2 kDa for R0.2, below 0.2 kDa for P0.2. [c]Treatments identified as in Table 1. [d] Total C recovered with the R0.2 and P0.2 fractions as % of total C recovered with all fractions.

Table S3. Chemical composition and property data for molecular weight fractions of SBPox described in Table 1 and above subsections 2.1-2.3: C and N content (w/w %), C/N w/w ratio, and surface tension (γ, mN m^{-1}) and colour for aqueous solutions containing 2 g L^{-1} added products.

**Treatment Nr-type[a]**	**Fraction**	**C, %**	**N, %**	**C/N**	**γ, mN m^{-1}**	**Color**
1-D0	R750	33.9	3.73	9.1		
	R150	41.5	4.93	8.4	69.0±0.047	Dark brown
	P0.2	38.9	4.67	8.3		
2-D 2	R750	25.1	2.12	11.9		
	R150	35.3	5.35	6.6	65.0±0.54	Dark brown
	P0.2	50.7	5.07	10.0		
3-D3	R150	44.2	5.59	7.9		
	R50	39.0	7.75	8.2		
	R20	44.3	5.14	8.6		
	R0.2	27.3	5.21	5.2		
	P0.2	22.4	6.73	3.3		
4-L0	R750	38.84	4.62	8.4		
	R150	37.71	4.45	8.5		
	R20	39.80	4.70	8.5	55.6±1.7	Beige
	R5	31.07	3.77	8.2		
	R0.2	32.54	4.16	7.8		
	P0.2	21.8	1.97	11.1		
5-L2	R150	36.5	5.33	6.9		
	R0.2	27.3	4.14	6.6		
	P0.2	20.4	1.51	13.6		
6-L3	R150	37.95	4.07	9.3		
	R20	14.26	0.56	25.7		
	R5	24.1	4.30	5.6		
	P0.2	21.4	4.84	4.4		

7-D0	R750	38.1	4.41	8.7	63.9±0.48	Dark brown
	R150	37.9	4.56	8.3	65.2±0.049	Dark brown
	R50	34.6	4.08	8.5		
	R20	40.3	4.90	8.2		
	R0.2	38.2	4.67	8.2		
	P0.2	14.2	0.59	25.0		
8-D2	R750	31.4	3.36	9.4		
	R150	35.2	4.52	7.8		
	R50	27.4	2.91	9.4		
	R20	38.0	5.22	7.3		
	R0.2	33.9	4.29	7.9		
	P0.2	13.3	0.68	19.7		
9-D3	R750	32.1	3.75	8.6	52.4±0.16	
	R20	32.4	4.24	7.6		
	R0.2	33.0	4.34	7.6		
	P0.2	13.2	0.79	17.3		
10-L0	R750	22.1	2.09	10.6		
	R150	30.7	3.46	9.0		
	R50	39.9	4.75	8.4	63.1±0.005	
	R20	39.2	4.69	8.4	64.0±0.45	
	R0.2	36.5	4.30	8.5		
	P0.2	10.1	0.31	32.4		
11-L2	R750	26.3	2.52	10.5		
	R50	31.3	3.92	8.0		
	R0.2	34.6	4.27	8.2		
	P0.2	15.0	1.11	13.5		
12-L3	R750	26.0	2.71	9.6	53.5±0.068	
	R20	34.3	4.39	7.8		
	R5	30.1	4.17	7.2		
	R0.2	31.6	4.04	7.8		
	P0.2	10.5	0.64	16.7		
13-D0	R750	42.4	6.13	6.9	54.1±0.22	Light brown
	R20	44.2	6.70	6.6		
	R0.2	36.6	6.86	5.3		
	P0.2	16.3	1.85	8.8		
14-D0.5	R20	41.9	4.57	9.2	45.9±0.056	Yellow
	R0.2	38.3	7.76	5.0		
	P0.2	16.5	1.70	9.7		
15-L0	R750	41.2	6.12	6.7	55.0±.0.008	Brown
	R150	39.9	6.66	6.0	57.4±0.01	Yellow
	R20	8.71	0.66	13.3		
	P0.2	10.3	0.21	49.0		
16-L0.5	R150	38.8	5.23	7.5	48.8±0.20	Pale yellow
	R20	40.3	5.53	7.3	48.3±0.029	Brown
	R5	33.7	4.95	6.8		
	P0.2	19.9	2.25	8.9		
17-D0.1	R750	43.1	5.25	8.2	51.0±0.12	Light brown
	R150	42.5	7.22	5.9		
	R50	41.9	7.13	5.9	59.0±0.83	Brown
	R5	40.2	7.08	5.7		
	P0.2	22.1	2.99	7.4		
18-D0.25	R750	41.3	5.11	8.1	49.4±0.12	Beige

	R150	37.6	9.31	4.0	54.6±0.89	Pale Yellow
	R50	41.8	8.56	4.9	64.1±0.17	Brown
	R20	41.4	7.75	5.3		
	R0.2	15.2	0.49	31.2		
	P0.2	19.9	2.58	7.7		
19-D0.5	R750	43.1	3.97	10.9	47.1±0.018	Pale yellow
	R150	42.0	6.46	6.5	62.7±0.50	Light browm
	R50	40.8	7.28	5.6	60.1±0.035	Beige
	R0.2	14.5	0.73	20.1		
	P0.2	10.7	0.55	19.4		

[a]Legends for treatments and fractions as in Table 1 and 3, and in Figure 1. Abbreviations for treatment 13 through 19 not reported in Table 1 have the same meaning: i.e.the treatment number followed by D or L indicates in the absence or presence of irradiation, respectively; the number after D and L indicats the added H_2O_2/C mole ratio.

Table S4. Surface tension values (γ, mN m^{-1}) in Table S3 and Figure 5 and mean values for $\gamma < 50$, $50 \leq \gamma < 60$, $\gamma \geq 60$ groups.

**Treat**[a]	**Ri**[b]	**$\gamma \geq 60$**	**Ri**[b]	**$50 \leq \gamma < 60$**	**Ri**[b]	**$\gamma < 50$**
1-D0	R150	69.0				
2-D0	R150	65.0				
4-L0			R20	55.6		
7-D0	R750	63.9				
7-D0	R150	65.2				
9-D3			R750	52.4		
10-L0	R50	63.1				
10-L0	R20	64.0				
12-L3			R750	53.5		
13-D0			R750	54.1		
14-D0.5					R20	45.9
15-L0			R750	55.0		
15-L0			R150	57.4		
16-L0.5					R150	48.8
16-L0.5					R20	48.3
17-D0.1			R750	51.0		
17-D0.1			R50	59.0		
18-D0.25	R50	64.1	R150	54.6	R750	49.4
19-D0.5	R150	62.7			R750	47.1
19-D0.5	R50	60.1				
N[c]		9		9		5
Mean[d]		64.1 a		54.7 b		47.9 c
Std[d]		2.4		2.6		1.4

[a]Treatment and [b]Retentate (Table 1 and 4). [c]Number of data points. [d]Values with different letters are significantly different at P <0.01 level; e.g., a > b > c. [e]Standard deviation.
